# Evolution of Surname Distribution under Gender-Equality Measures

**DOI:** 10.1371/journal.pone.0018105

**Published:** 2011-04-14

**Authors:** Luis F. Lafuerza, Raul Toral

**Affiliations:** IFISC (Instituto de Fsica Interdisciplinar y Sistemas Complejos), CSIC-UIB, Campus UIB, Palma de Mallorca, Spain; University of Zaragoza, Spain

## Abstract

We consider a model for the evolution of surname distribution under a gender-equality measure currently being discussed by the Spanish Parliament (whereby children would adopt their mother's and father's surnames in alphabetical order). We quantify how this would bias the alphabetical distribution of surnames, and analyze its effect on the present distribution of surnames in Spain.

## Introduction

In Spain, as in many other countries, children usually inherit their father's surname. As a consequence, the mother's surname is lost in the child's generation (in Spain, however, the mother's surname is kept as a second surname, it is consequently totally lost in the grand-children's generation). Nowadays, in Spain, parents can agree upon whether it is the mother's or father's surname that is given to their children, but if parents do not reach an agreement, it will automatically be the father's surname that is inherited by the children. Due to gender-equality issues, a new law is under review whereby, if either, parents do not reach an agreement, or if no wish is expressed, the surname inherited by the children will be selected according to the alphabetical order of the parents' two surnames.

People have immediately realized that this implies a bias on the surnames favoring those beginning with the first letters in the alphabet (A,B,…) and could mean that surnames beginning with the last letters (…,Y,Z) disappear completely. In this short paper, we quantify the effect of this bias on the present distribution of surnames in Spain.

Evolution of surnames distribution (in the absence of any alphabetical preference) has been studied previously. The pioneering work of Galton and Watson used a branching process to study the probability of extinction of surnames in English aristocracy[1]. This problem turns out to be mathematically equivalent to that of the evolution of non-recombining neutral alleles [2] and several authors[3]–[5] have used similar ideas in the context of biological evolution. Later developments by mathematicians and physicists centered on the distribution of family sizes [6]–[9]. The novelty of our work is that we analyze how the distribution of surnames evolves when a preference depending on their alphabetical position is present. We derive an equation for the evolution of surnames distribution on these premises. The solution of the equations allows us to determine the timescale at which the surnames disappear.

## Analysis

As a first order model aimed at capturing the essence of the process of surname inheritance we propose the following:


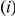
 Initially, a population of 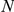 individuals (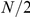 male and 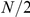 female) is considered. Each individual has a surname chosen according to some prescribed distribution.


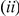
 Males and females reproduce in random pairs in such a way that, on average, the total population remains constant.


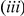
 With probability 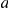 it is assumed that parents reach an agreement, so that the surnames of their children are chosen at random between those of the parents (the proportion of whether the father's surname or the mother's is preferred is irrelevant on the results). With probability 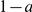, parents do not reach or do not express an agreement, and children adopt their surname by the alphabetical order rule.

We measure time 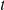 in average reproductions per person, or generations. In a generation, parents are replaced by their children in the population.

The population evolves according to a bisexual Galton and Watson branching process[10]. The statistics of the number of people as a function of time [11] and the distribution of the frequency of surnames in a model similar to this one when the surnames are chosen at random have been studied previously[8].

The model introduced above is a minimal model and does not consider some realistic issues such as geographical distribution of surnames, etc. but those are expected to be second order effects with little impact in the overall trend. The effect of immigration and population growth is analyzed later in the text.

Let us define 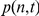 as the proportion of individuals (both males and females) with surname in the alphabetical position 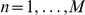, being 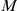 the total number of surnames. It evolves according to:
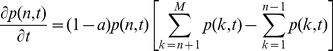
(1)

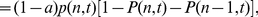
(2)where 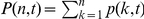 is the cumulative distribution. The first term in the square brackets of Eq.(1) represents the increase in probability of surname 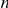 due to the pairing with surnames 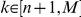 which are further forwards the end in the alphabetical order, while the second term represents the loss in probability due to pairings with surnames 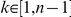 earlier in the alphabetical order. It follows that:
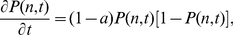
(3)whose solution is:
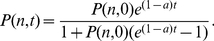
(4)


The distribution of surnames at time 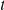 is then 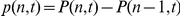 for 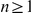 with the convention 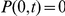. Approximating the difference by a derivative 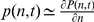, we obtain:
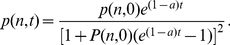
(5)


Eq. (4) shows that the distribution of surnames approaches a Kronecker-delta at 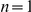 (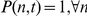) exponentially quickly with a characteristic time 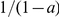. Assuming, for instance, that couples reach, and express, an agreement in 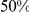 of the cases (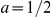), we find from Eq.(5) that the frequency of a surname towards the end of the alphabetical table would be decreased by a factor 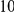 in around 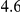 generations(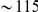 years). If, on the other hand, couples do not reach an agreement in 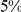 of the cases (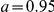), then the decrease by a factor 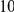 occurs in 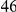 generations.

### Evolution of current distribution

**Figure 1:**
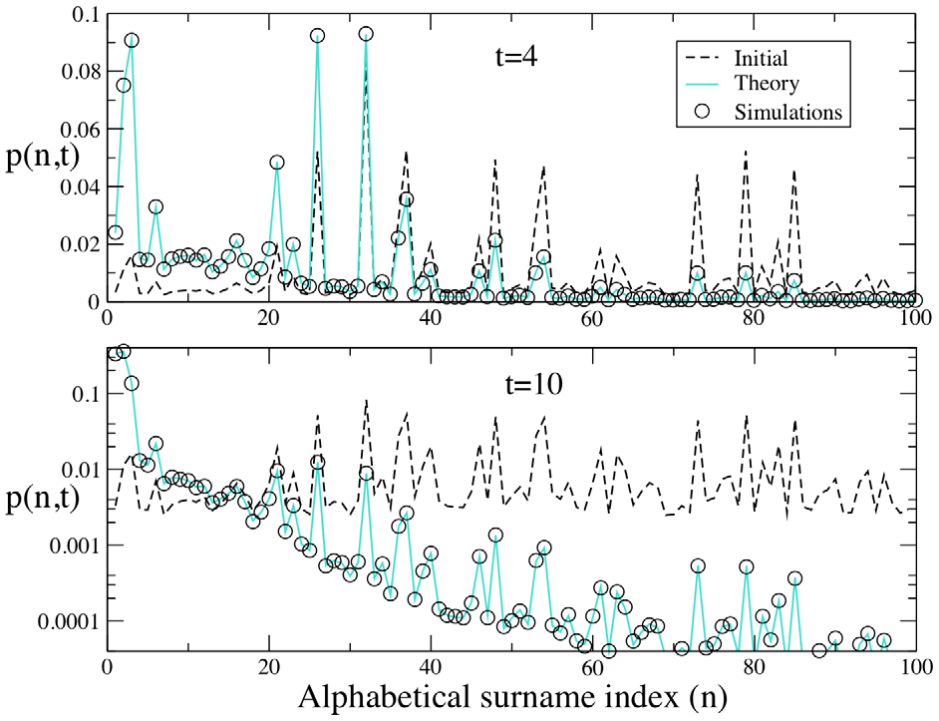
Evolution of the distribution of surnames after 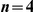 (top) and 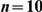 (bottom) generations, taking as initial condition 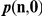 the actual distribution of the 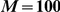 most common surnames in Spain. For 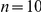 we have used a logarithmic scale for a better viewing of the data. The dots are the result of the numerical simulation of the model described in the main text, and solid lines correspond to the analytical result (4).

We have applied the above results to the current distribution of Spanish surnames. Besides the analytical result of Eq.(5), we have performed a numerical simulation of the model by which 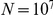 couples have probabilities 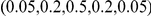 of having 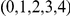 children (average value is 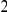). The probability of parents reaching an agreement is set at 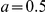. Whether an agreement has been reached or not, the rule applied to the first-born child is used for all subsequent children. We have used as the initial condition 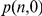 the distribution of the 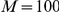 most common surnames in Spain, after ordering them in alphabetical order. The data appear in the INE webpage www.ine.es (INE stands for “Instituto Nacional de Estadística”). Similar data is available for other countries. Our simulation results only consider those 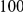 surnames for which data are publicly available. In figure 1 we plot (symbols) the probability distribution 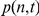 resulting from this numerical simulation after 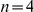 (top panel) and 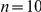 (bottom panel) generations. In the same figure we also plot the theoretical prediction, Eq. (5) using the same initial condition and for the same number of generations. As it can be seen in the figure (note the logarithmic scale for better viewing of data in the case 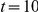) the concurrence between the simulation and the analytical result is excellent. It can be noted that the relative importance of surnames moves towards the surnames which are earlier in the alphabet as time increases.

The evolution in the frequency of a surname does not have to be monotonous, as it can first increase and then decrease in time. Let us take, for instance, the most common surname in Spain: “Garca”. According to the INE data, there are 1,481,923 people bearing this surname and the cumulative distribution is 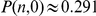. Hence, if there is a 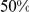 agreement, the frequency or this surname would first increase up to 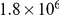 in two generations, to then decrease to 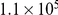 in 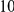 generations. In the case of the surname “Toral”, there are nowadays 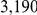 people bearing this surname and the cumulative distribution is 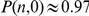. According to the previous analysis, and considering again 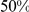 agreement, it would decrease to 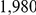 in one generation and to 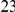 in 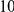 generations. The same study for “Lafuerza” (very rare surname, only 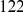 people bear it in Spain currently and the cumulative distribution is 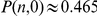), shows that it would stay practically constant in the first generation to then decrease to 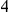 (practical extinction) in 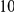 generations. Finally, if we take a surname high in the alphabetical order such as “Aguilar”, of which there are 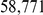 people at the moment, it would increase practically exponentially, as the cumulative distribution is very small and can be neglected in the denominator of Eq.(5). Of course, all these predictions are for the mean values, and significant statistical deviations could occur for low-frequency surnames.

### Effect of immigration and population growth

In the previous analysis we assumed that the population remains constant (there is an average of two children per couple) and that the only changes in the distribution of surnames correspond to the application of the alphabetical order rule. We now consider the effect that, both, population growth and new surnames brought in by immigration have in the distribution of surnames. This implies modifying condition 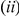 in the model, allowing the number of children per couple to have any average value 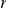 and setting immigration events with rate 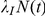, proportional to the total population number. The alphabetical position of the surname of the immigrant is chosen at random according to some probability distribution 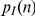. The total population increases exponentially as 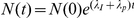 with 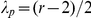.

Let 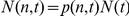 be the number of people with surname in alphabetical position 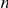. It evolves according to:
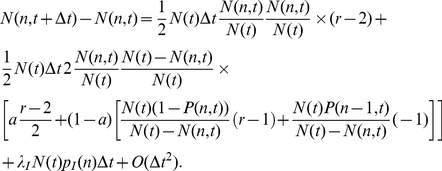
(6)


The first term corresponds to mating of two people (one male, one female) both with surname in alphabetical position 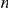, in this case the average increase in 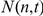 equals the average increase due to the mating, which is 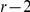. The second term corresponds to the mating of a person with surname 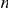 with a person with a different surname: if, with probability 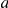, they agree on the surname to be assigned to the children, the average increase in 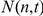 equals 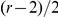; otherwise, the average increase in 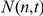 is either 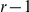 (if the other surname is later in the alphabet) or 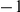 (if it is earlier). This derivation neglects the possible fluctuations that can appear in the distributions of surnames in males and females. The last term corresponds to the addition of new individuals brought in by immigration.

From this equation, and after some algebra, one can obtain the evolution of the number of people 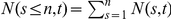 with surname in alphabetical position smaller or equal to 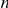:
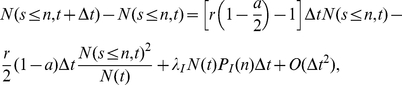
(7)being 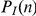 the cumulative distribution of the immigrants surnames. Dividing by 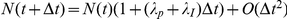 and taking the limit 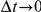, we obtain:
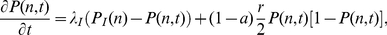
(8)whose solution is:
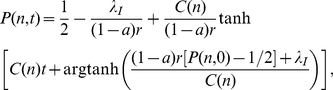
(9)with 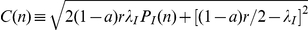.

We see from Eq.(8) that the intrinsic growth, 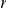, of the population only changes the timescale of the dynamics of the surname distribution. Immigration, however, might have a greater impact. Let us focus on the asymptotic, 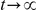 distribution, which has the form:
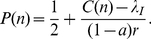
(10)


An analysis of this expression, shows that a critical value 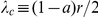 exists, such that the delta-like singularity that appeared in the non-immigration case disappears for 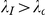. The situation then, is that for 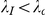 there is an accumulation at surnames close to the lower limit 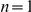, such that a fraction 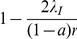 of people bear the surname first in the alphabetical order. So, the low immigration rate produces results which are the same, qualitatively, as in the case without immigration. For 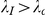, however, the fresh distribution of surnames brought in by immigration is enough to overcome the accumulation at 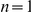 towards which the probability distribution would tend in the asymptotic limit due to the alphabetical order rule. In both cases, the tail of the stationary probability distribution, behaves as 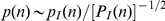. If, for instance, the distribution of new surnames were uniform in the alphabet, 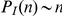, then the tail of the stationary distribution would behave as a power-law of slope 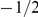.

## Discussion

We have developed a mathematical model for the evolution of the surnames distribution when an alphabetical-order rule on the progenitor's surnames is applied. The premises of the model lead to a differential equation governing the evolution of the probability distribution. As initial condition we have considered the data for the present distribution of the 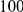 most common surnames in Spain, obtained from the National Statistics Institute of Spain (INE), that is publicly available at its webpage www.ine.es. Similar data is available for other countries. We have also performed numerical simulations of an agent-based model, which agree with the analytical result.

In our minimal model for surname transmission, we prove that the adoption of the alphabetical rule leads to an exponential decrease in the surnames that begin with letters that are towards the end of the alphabet, with a characteristic decay time of 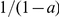 generations, being 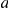 the fraction of parents that reach an agreement. This quantifies the decrease in the frequency of those surnames. We have also considered the effect of surnames brought in by immigration and found that, below a critical value of the immigration rate, the results are the same, qualitatively, as in the case without immigration. For large immigration rates, the delta-like singularity that appeared at names earlier in the alphabet, disappears.

We believe that this study offers an example in which statistical methods and mathematical modeling can be used to quantitatively calculate the consequences of a political measure and, consequently, it can serve as a guide to institutions and policy makers.
